# Depolarization Ratio of the ν_1_ Raman Band of Pure CH_4_ and Perturbed by N_2_ and CO_2_

**DOI:** 10.3390/molecules27010144

**Published:** 2021-12-27

**Authors:** Aleksandr S. Tanichev, Dmitry V. Petrov

**Affiliations:** 1 Laboratory of Ecological Instrumentation, Institute of Monitoring of Climatic and Ecological Systems, Siberian Branch of the Russian Academy of Sciences, 634055 Tomsk, Russia; dpetrov@imces.ru; 2 Department of Optics and Spectroscopy, Tomsk State University, 634050 Tomsk, Russia

**Keywords:** Raman spectroscopy, depolarization ratio, ν_1_ band, methane, nitrogen, carbon dioxide

## Abstract

In this work, the effect of nitrogen and carbon dioxide on the depolarization ratio of the ν_1_ band of methane in the pressure range of 0.1–5 MPa is studied. A high-sensitivity single-pass Raman spectrometer was used to obtain accurate results. Moreover, we took into account the overlap of the ν_1_ band by the ν_3_ and ν_2_ + ν_4_ bands using the simulation of their spectra. The depolarization ratio of the ν_1_ band in pure methane is within 0–0.001, and the effect of nitrogen and carbon dioxide on this parameter is negligible in the indicated pressure range. The obtained results are useful for correct simulation of the Raman spectrum of methane at different pressures, which is necessary to improve the accuracy of gas analysis methods using Raman spectroscopy.

## 1. Introduction

The optical methods based on Raman spectroscopy for the analysis of multicomponent gaseous media have been rapidly developing in the last decades. These methods can simultaneously detect all molecular vibrational bands using one laser with a fixed wavelength. The recent appearance of high-sensitivity photodetectors and powerful small-size lasers provides an opportunity to amplify the useful signal and decrease the limit of detection (LOD) of the method. Other amplification methods include the multi-pass optical cells [1,2,3,4] or hollow-core fiber [5,6,7,8]. However, compression of the analyzed medium to a higher pressure is the most effective and easy to implement signal amplification approach [9,10]. Neglecting the compressibility factor, compression of the sample at ambient pressure to a pressure of 5 MPa leads to a 50-fold amplification [11]. The LOD below 1 ppm can be achieved using this approach. Such sensitivity opens up the possibility to analyze the composition of atmospheric and exhaled air using Raman spectroscopy [5,10,12,13,14,15,16]. This method is very promising due to its high measurement speed and the ability to determine a lot of compounds.

Methane (CH_4_) is an important greenhouse gas contained in atmospheric air. The average annual concentration of CH_4_ is continuously increasing due to the influence of natural and anthropogenic factors. Therefore, monitoring of atmospheric CH_4_ is necessary to detect leaks of greenhouse gases, as well as to improve climate prediction models. The measurement precision of concentration should be less than 100 ppb since CH_4_ content in atmospheric air is about 2 ppm [17]. Moreover, CH_4_ is included in the list of biomarkers of diseases [18,19]. The CH_4_ content in the exhaled air can reach 10 ppm [12]. Hence, high accuracy of CH_4_ measurement in a sample is necessary both for the accurate diagnosis and for the investigation of correlations. On the other hand, the region of stretching C–H vibrations (2900–3000 cm^−1^) is important in the air composition analysis since the most intense vibrational Raman bands of all volatile organic compounds (VOCs) are located there [20,21]. The CH_4_ content in the air is high compared to other VOCs. Therefore, the most intense fundamental vibrational ν_1_ band of CH_4_ (≈2917 cm^−1^) makes a significant contribution to the spectrum of these gaseous media in the indicated spectral range. It should be noted that pressure and different molecular environment significantly affect the spectroscopic characteristics of the ν_1_ peak of CH_4_ [22,23,24,25,26,27,28,29,30]. The higher the molar fraction of the perturbing component, the stronger its influence on the spectrum. The effect of the main components of air cannot be neglected since their concentration is orders of magnitude higher than CH_4_. Thus, it is necessary to account for these changes in the CH_4_ spectrum to decrease the LOD of CH_4_ and correctly derive the concentrations of other organic compounds from the Raman spectra of air. A simulation method of the CH_4_ spectrum is one of the most promising approaches for this purpose [26,31,32,33]. The required intensities and positions of spectral lines can be obtained using calculations based on the tensor formalism and the group theory methods [34]. However, it is necessary to know the tensor components of the total polarizability derivative of the molecule. The depolarization ratio (*ρ*) can be expressed in terms of these quantities [35,36,37]. Since the spectral sensitivity of a spectrometer is not the same for radiation in different polarization states, the use of the exact experimental value of the *ρ* can both increase the reliability of theoretical calculations and more accurately fit the simulated spectra to the experimental ones.

As noted by Wang and Ziegler [38], the depolarization ratio of the ν_1_ band of CH_4_ (*ρ*(ν_1_)) is 0.025 ± 0.005 at a pressure of less than 0.1 MPa. More precise measurements were performed using the photoacoustic Raman spectroscopy method by Yu et al. [39], where the *ρ*(ν_1_) = 0.002 ± 0.002 was obtained at a pressure of less than 15 kPa. According to the data reported in [40,41,42], the *ρ*(ν_1_) is a function of pressure. However, there is a discrepancy between the results obtained, for example, *ρ*(ν_1_) = 0.11 at 4 MPa [40], *ρ*(ν_1_) = 0.067 at 5 MPa [41], and *ρ*(ν_1_) = 0.0045 at 6 MPa [42]. Moreover, as shown in [40,41,43,44,45], the molecular environments also affect the *ρ*(ν_1_). Taking into account the composition of air, knowledge about the influence of nitrogen (N_2_) and carbon dioxide (CO_2_) on this parameter is important in the field of Raman analysis of atmospheric and exhaled air. However, to our knowledge, no studies have investigated the N_2_ environment effect. The *ρ*(ν_1_) perturbed by CO_2_ was measured with a very high error [43]. We suppose that this error is due to the low signal-to-noise ratio of the equipment used. In this work, the influence of the N_2_ and CO_2_ environments on the *ρ*(ν_1_) of CH_4_ in the pressure range of 0.1–5 MPa at 298 K was researched. A high-sensitivity Raman spectrometer and a simulation of the CH_4_ spectrum were used for this purpose.

## 2. Methods

**Figure 1:**
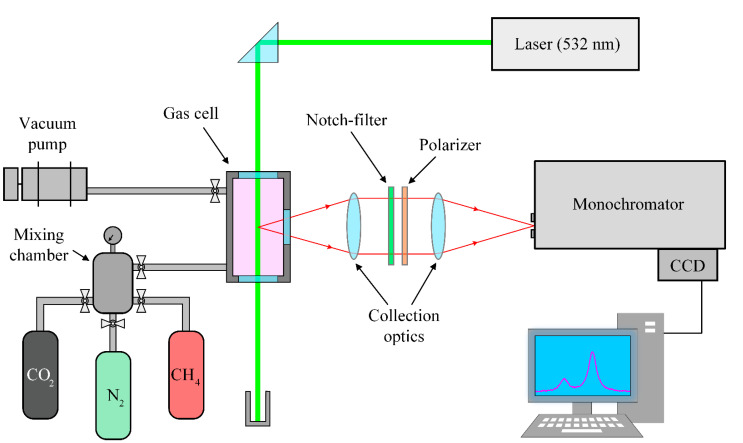
Schematic of the experimental setup.

Despite the recent advances in the field of amplification of the Raman signal [1,2,3,4], the experimental setup based on the single-pass excitation scheme was used to obtain reliable data in this study. Figure 1 and Table 1 present the scheme and the main characteristics of the setup, respectively. Plane-polarized radiation of the single-mode continuous-wave laser (Cnilaser, Changchun, China) with a wavelength of 532 nm was directed into the gas cell and excited spontaneous Raman scattering in the medium. The scattered radiation was collected at an angle of 90° to the direction of propagation of the laser beam through the side window of the gas cell using the system of two lenses. The notch filter and the polarizer were installed between them. Polarized radiation was focused on the entrance slit of the spectrometer based on the Čzerny–Turner configuration. The Raman spectra were recorded using the charge-coupled device (CCD) sensor S10141 (Hamamatsu Photonics K.K., Hamamatsu, Japan) with thermoelectric cooling to −10 °C. The simultaneously recorded spectral range was 2800–3040 cm^−1^.

Polarized and depolarized spectra of pure CH_4_, as well as mixtures of CH_4_/N_2_ and CH_4_/CO_2_ in molar ratios of 50/50, at pressures of 0.1, 0.5, 1, 2, 3, 4, and 5 MPa were recorded using this system. The signal-to-noise ratio in the polarized spectra of pure CH_4_ was 1500 (at 0.1 MPa) and 11,000 (at 5 MPa), where the peak intensity of the ν_1_ band (≈2917 cm^−1^) was the signal magnitude. The pressure measurement error was less than 1 kPa. The gas cell was thermally stabilized at 298 ± 1 K. Samples of CH_4_, N_2_, and CO_2_ with a purity of greater than 99.99% were used to prepare the studied mixtures in a separate mixing chamber connected to a gas cell. Pure gases were mixed in a specified ratio of partial pressures to obtain the required molar ratio. These partial pressures were calculated from the equation of state for gases taking into account the compressibility. Compressibility factors were taken from the NIST Chemistry WebBook [46]. The molar ratio measurement error in the mixture preparation procedure is estimated within 2–3%.

The wavenumber calibration of the spectrometer was performed using the spectrum of pure CH_4_ at a pressure of 0.1 MPa according to the procedure described by Brunsgaard Hansen [47]. However, the most intense lines of the ν_3_ band from data of Berger [48] were taken as reference lines, instead of the emission lines of a neon lamp. As a result, the third-degree polynomial was obtained, representing the relationship between the pixel numbers of the CCD sensor and the wavenumbers of the spectrometer. The calibration error and the spectrum drift due to ambient temperature fluctuations were estimated to be less than 0.02 cm^−1^.

## 3. Results and Discussion

### 3.1. Raman Spectra of Methane

**Figure 2:**
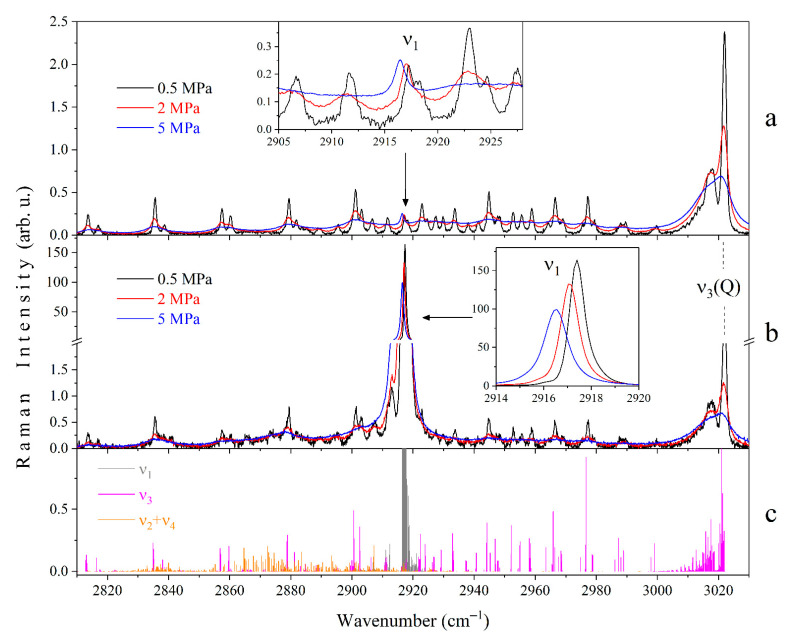
Experimental depolarized (**a**) and polarized (**b**) Raman spectra of pure CH_4_ at pressures of 0.5, 2, and 5 MPa. The spectra are normalized by the integrated intensity of the Q branch of the ν_3_ band. The insets show the effect of pressure on the ν_1_ peak. Panel (**c**) shows the positions and intensities of the rotational-vibrational lines of the ν_1_, ν_3_, and ν_2_ + ν_4_ bands calculated by Ba et al. [52].

**Figure 3:**
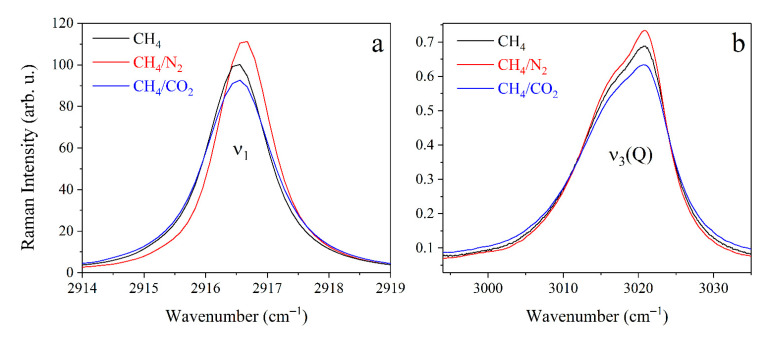
Experimental Raman spectra of the polarized ν_1_ band (**a**) and the depolarized ν_3_ band (**b**) of pure CH_4_ and CH_4_/N_2_ and CH_4_/CO_2_ mixtures at a pressure of 5 MPa. All spectra were normalized by the integrated intensity.

Figure 2 shows the obtained Raman spectra of pure CH_4_ at various pressures in the spectral range of 2810–3030 cm^−1^. The polarized spectrum is the high-intensity peak formed by closely spaced rotational-vibrational lines of the Q branch of the ν_1_ band. This peak is overlapped by the O, P, and Q branches of the ν_3_ band and the Q branch of the ν_2_ + ν_4_ band. The contribution of other overtones and hot transitions can be neglected in this range. The vibrations ν_1_ and ν_2_ + ν_4_ are characterized by extremely weak anisotropic polarizability properties. Hereby, the ν_2_ + ν_4_ band is not observed in the depolarized spectra, and the ν_1_ band is a low-intensity peak. An increase in medium pressure leads to the broadening of all lines due to the collisional broadening effect. Therefore, the ν_3_ band is an almost continuous spectrum at a pressure of 5 MPa. However, this effect is not so pronounced for the ν_1_ band, since the processes of collisional line mixing dominate here [26]. The ν_1_ peak shifts to the region of low wavenumbers as the pressure increases, which corresponds to the data of [22,27,28,49,50]. The effect of the N_2_ and CO_2_ environments leads to different broadening and shifts of the CH_4_ lines. Nevertheless, the spectrum of the mixture is similar to that of pure CH_4_ at a different pressure. This difference is more pronounced as the pressure increases. As shown in Figure 3, the presence of N_2_ in the mixture leads to a narrowing of the ν_1_ peak, while the presence of CO_2_ leads to a broadening. It is also worth noting that the N_2_ environment leads to a smaller shift of the ν_1_ peak to the region of low wavenumbers than CH_4_ or CO_2_. These observations are in agreement with results presented in [22,27,51]. The contribution of the N_2_ and CO_2_ bands is negligible within the spectral range under investigation in comparison with the ν_3_ and ν_2_ + ν_4_ lines.

### 3.2. Measurement Procedure

**Figure 4:**
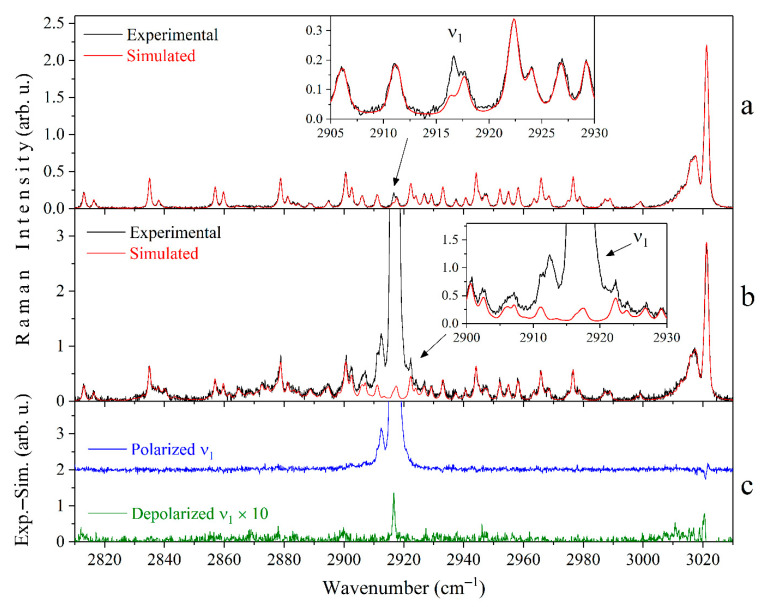
Subtraction procedure of the simulated spectra from the depolarized (**a**) and polarized (**b**) experimental Raman spectra of pure CH_4_ at a pressure of 0.5 MPa. The spectra are normalized by the integrated intensity of the Q branch of the ν_3_ band. Panel (**c**) shows the obtained differences, where the depolarized spectrum is magnified 10 times for visualization.

The observed depolarization ratio of an arbitrary vibrational band can be defined by Equation (1),

(1) $$\rho =\frac{\int_{\omega }E^{⊥}\left(\omega \right)d\omega }{\int_{\omega }E^{∥}\left(\omega \right)d\omega },$$

where $E^{⊥}\left(\omega \right)$ and $E^{∥}\left(\omega \right)$ are the intensities of the experimental Raman spectra at the wavenumber *ω*, when the polarization planes of the scattered and exciting radiation are parallel (polarized spectrum) and perpendicular (depolarized spectrum), respectively. Here, it is necessary to take into account the overlap of the ν_3_ and ν_2_ + ν_4_ bands at different pressures and environments to correctly measure the integrated intensity of the ν_1_ band. The method of simulating the Raman spectrum as a sum of the profiles of each rotational-vibrational line was used for this purpose. A detailed description of this approach can be found in our previous work [33]. The positions and intensities of the ν_3_ and ν_2_ + ν_4_ lines were taken from the study of Ba et al. [52], and the pressure broadening and shift coefficients were used the same as those in [33]. According to the features of the polarizability anisotropy, only the ν_3_ lines were used to simulate the depolarized spectra. The ν_3_ and ν_2_ + ν_4_ lines were used to simulate the polarized spectra. The influence of the N_2_ and CO_2_ environments on the ν_3_ and ν_2_ + ν_4_ bands of CH_4_ was imitated by simulating the spectrum at a different pressure. The integrated intensities of the depolarized and the polarized ν_1_ band ($E^{⊥}\left(\nu_{1}\right)$, $E^{∥}\left(\nu_{1}\right)$) were measured in the range of 2880–2950 cm^−1^ in each experimental spectrum after subtracting the simulated spectrum (see Figure 4).

**Figure 5:**
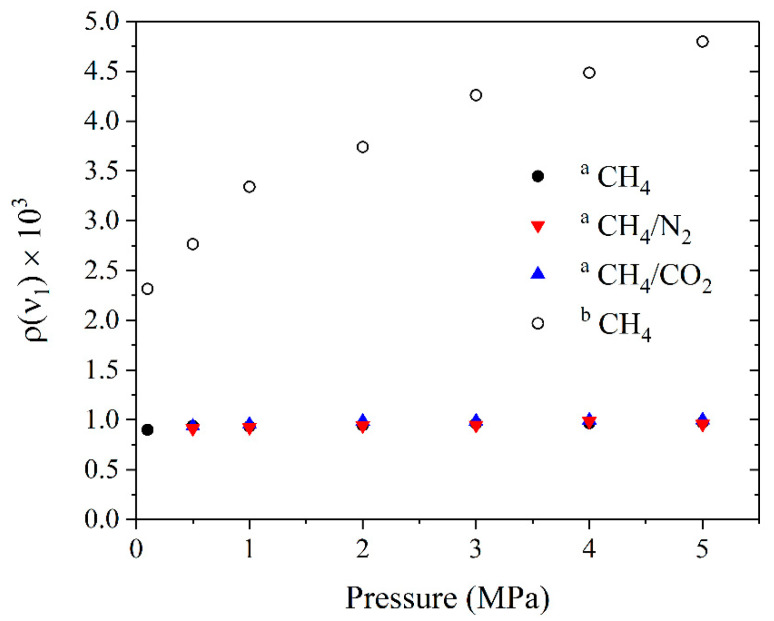
Depolarization ratio of the ν_1_ band of CH_4_ as a function of pressure at different molecular environments, where label **^a^** denotes the data obtained after the subtraction procedure of the simulated spectrum of the ν_3_ and ν_2_ + ν_4_ bands, and label **^b^** is the data obtained without the subtraction.

Further, it is necessary to take into account the fluctuations of the laser power to improve the accuracy of the intensity measurement. We used the Q branch of the ν_3_ band of CH_4_ for this purpose since the *ρ* of this band does not depend on pressure in the range of 0–5 MPa and equals 0.75 [42,53]. Thus, the *ρ*(ν_1_) values were obtained using Equations (2) and (3):

(2) $$\rho \left(\nu_{1}\right)=\frac{E^{⊥}\left(\nu_{1}\right)}{E^{∥}\left(\nu_{1}\right)}k,$$

(3) $$k=0.75\frac{E^{∥}\left(\nu_{3}\right)}{E^{⊥}\left(\nu_{3}\right)},$$

where $E^{⊥}\left(\nu_{3}\right)$ and $E^{∥}\left(\nu_{3}\right)$ are the integrated intensities of the Q branch of the ν_3_ band in the depolarized and polarized spectra, respectively. These intensities were measured in the range of 3000–3030 cm^−1^. The data obtained are presented in Figure 5. The values of the *ρ*(ν_1_) are in the range of 0.0009–0.001 and the influence of the molecular environment in the pressure range of 0–5 MPa is not observed. The double standard deviation of all measurements is less than 0.0001. It should be noted that much larger values of the *ρ*(ν_1_) at 5 MPa were obtained by other authors [40,41,42]. We suppose that this discrepancy is caused by the neglect or incorrect accounting of the overlap of the ν_1_ peak by the ν_3_ and ν_2_ + ν_4_ bands, in addition to the low signal-to-noise ratio. The obtained values of the *ρ*(ν_1_) of pure CH_4_, where the subtraction procedure of the simulated spectra was not performed, are also shown in Figure 5 for comparison. It can be seen that the pressure dependence of the *ρ*(ν_1_) is observed in this case, which corresponds to the previous results [42]. The reason for this is that the contribution of the ν_3_ and ν_2_ + ν_4_ lines to the intensity of the ν_1_ band (in the 2910–2925 cm^−1^ range) increases due to the collisional broadening effect. Thus, the data in Figure 5 confirm that the contribution of depolarized lines must be taken into account to obtain the most reliable values of the *ρ*.

### 3.3. Uncertainty Evaluation

**Figure 6:**
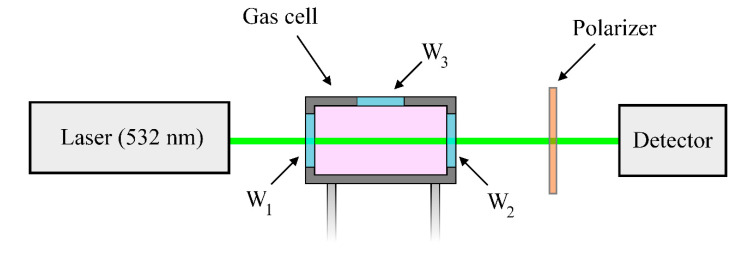
Schematic of the experimental setup used to evaluate the effect of polarization scrambling. Here, W_1_, W_2_, and W_3_ denote the windows of the gas cell.

According to Figure 5, the measured value of the *ρ*(ν_1_) is not equal to zero even at a pressure of 0.1 MPa, which does not agree with theoretical calculations [35,53,54]. Let us estimate the error of our measurements. The main sources of the measurement error are imperfect polarization of the laser radiation, different transmittance of the polarizer in orthogonal orientations, and polarization scrambling by the windows of the gas cell [55,56], as well as the non-zero collection angle for the scattered radiation [38,57,58,59]. The additional experiment was carried out to evaluate the influence of the first three effects. Laser radiation was directed through the cell windows and the polarizer and was guided to the photodetector at the output (see Figure 6). At the first stage, the cell was pressurized by pure CH_4_ at 0.1 MPa and the power of the transmitted radiation was measured in two orthogonal polarization orientations. At the second stage, the pressure of CH_4_ in the cell was increased to 5 MPa and similar measurements were performed. It was found that the ratio $P^{∥}/P^{⊥}$ was more than 1000 in both cases, where $P^{⊥}$ and $P^{∥}$ are the measured radiation power with perpendicular and parallel orientation of the polarization plane to the polarization plane of the exciting radiation, respectively. It should be noted that the entrance window (W_1_) and the exit window (W_2_) influenced the results obtained in this experiment, but the window W_1_ and the side window (W_3_) influenced the measurements of the *ρ*(ν_1_). Since all the cell windows are identical, we can conclude that the systematic measurement error of the *ρ*(ν_1_) is less than 0.001 at a zero-collection angle, taking into account the aforementioned effects of polarization scrambling.

The approach based on the calculations presented by Schlösser et al. [57] was used to estimate the measurement error in the case of the non-zero collection angle. A detailed description of the calculations performed is provided in Appendix A of this study. As a result, the geometric effect introduces the systematic measurement error of no more than 2% of the *ρ*(ν_1_) = 0.001, without taking into account the effects of polarization scrambling. Since the non-zero angle effect has a small contribution, the estimate of the total systematic measurement error of the *ρ*(ν_1_) is less than 0.001. Therefore, we can conclude that the true depolarization ratio of the ν_1_ band of CH_4_ is within 0–0.001 in the pressure range of 0.1–5 MPa.

## 4. Conclusions

In this study, the depolarization ratio of the ν_1_ band of CH_4_ was measured using the Raman spectrometer that combines both high resolution and high sensitivity. It was found that the depolarization ratio of the ν_1_ peak of pure CH_4_ or perturbed by the N_2_/CO_2_ molecular environment did not exceed 0.001 in the pressure range of 0.1–5 MPa. This value is significantly less than the measurements reported in earlier studies. In our view, this discrepancy is a consequence of correctly taking into account the overlap of the ν_1_ band by the ν_3_ and ν_2_ + ν_4_ bands using the spectra simulation in this study. These results imply that the correction of the tensor components of the total polarizability derivative of CH_4_ due to the effect of the N_2_/CO_2_ environment, and pressure can be neglected in the pressure range of 0.1–5 MPa. Therefore, the line intensities of CH_4_ in vacuum calculated using the tensor formalism approach are suitable for simulating its spectra in the field of Raman gas analysis of methane-bearing media (e.g., fuel gases, atmospheric air, exhaled air, etc.).

## Figures and Tables

**Table 1:** Characteristics of the experimental setup.

Parameter	Unit	Quantity
Laser output power	W	5
Laser wavelength	nm	532.094
Polarization ratio	unitless	>100:1
Collection lens diameter (*D*_1_)/focal length (*F*_1_)	mm	26.3/105
Focusing lens diameter (*D*_2_)/focal length (*F*_2_)	mm	46.7/210
Distance between lenses (*d*)	mm	250
Spectrometer f-number	unitless	f/8
Size of CCD chip	pixel	2048 × 512
Diffraction grating	line/mm	2400
Entrance slit height (*h*_2_)/width	mm	4/0.03
Half-width of instrument response function	cm^−1^	0.5 (at 2917 cm^−1^)
Spectral dispersion	cm^−1^/pixel	0.12

**Figure A1:**
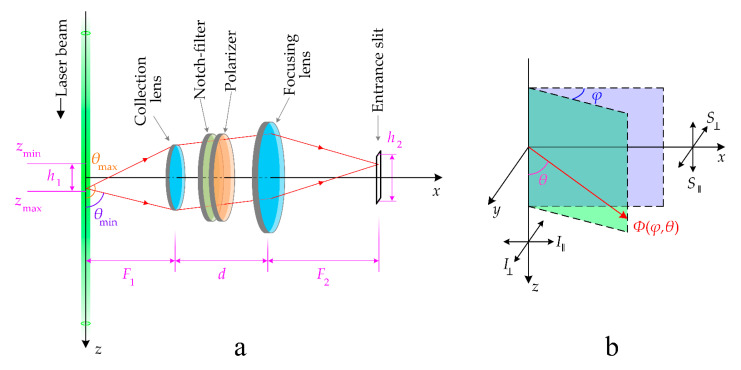
Sketch (**a**) is the optical collection system of the scattered radiation. The laser beam propagates along the z-axis, the scattered radiation is collected along the x-axis. Here, the path of two light rays from an arbitrary point z at an angle *φ* = 0 is shown. Sketch (**b**) is the definition of scattering angles, where $I_{⊥}$ and $I_{∥}$ denote the polarization directions of the incident radiation, $S_{⊥}$ and $S_{∥}$ denote the polarization planes of the scattered radiation, *φ* is the angle between the scattering plane (green) and the plane $S_{∥}$ (blue), and *θ* is the angle between the z-axis and the direction of scattering *Φ*(*φ,θ*) in the scattering plane.

**Figure A2:**
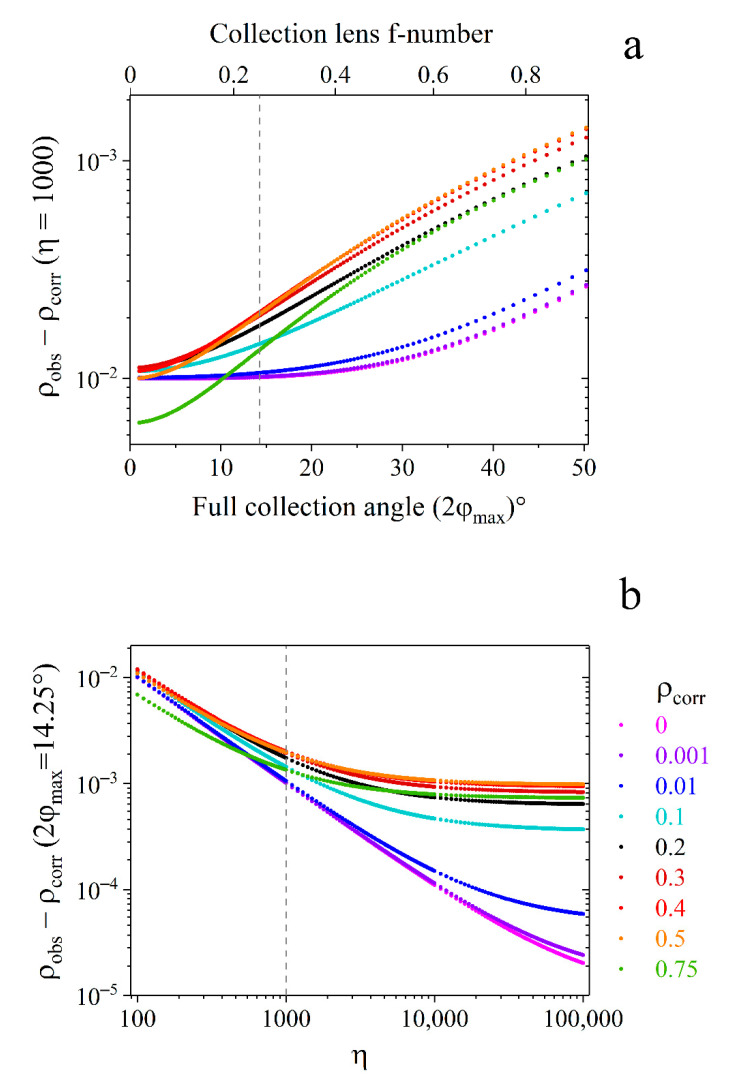
Deviation of the observed depolarization ratio from the corrected one as a function of the total collection angle 2*φ*_max_ (**a**) and the parameter *η* (**b**), calculated using Equations (A13) and (A16)–(A25). The dashed line denotes the boundary values of the angle 2*φ*_max_ = 14.25° (lens f/4) and the parameter *η* = 1000, corresponding to the experimental setup used in this work.

## Data Availability

Data are contained within the article.

## Appendix A

The integrated intensity of an arbitrary vibrational Raman band *v_j→k_* observed in the direction (*φ*,*θ*) can be expressed as follows [60]:

(A1) $$E_{obs}(\varphi ,\theta )∼E_{in}N_{j}g_{j}\Phi_{j}(S_{p},I_{p},\varphi ,\theta ),$$

where *E_in_* is the intensity of the incident radiation exciting the Raman scattering; *Φ_j_* is the scattering strength function of the scattered radiation in the direction (*φ*,*θ*); *φ* and *θ* are the scattering angles; *N_j_* and *g_j_* are the population and the degeneracy of the *j*th vibrational energy level; and *I_p_* and *S_p_* are the polarization states of the plane-polarized incident and scattered radiation (*p* = ⊥ or ∥), respectively (see Figure A1 for details). The combinations of the polarization states assign the four scattering strength functions:

(A2) $$\Phi (S_{⊥},I_{⊥},\varphi ,\theta )=45\alpha^{2}\cos^{2}\varphi +\gamma^{2}\left(3+\cos^{2}\varphi \right),$$

(A3) $$\Phi (S_{∥},I_{⊥},\varphi ,\theta )=45\alpha^{2}\cos^{2}\theta \sin^{2}\varphi +\gamma^{2}\left(3+\cos^{2}\theta \sin^{2}\varphi \right),$$

(A4) $$\Phi (S_{⊥},I_{∥},\varphi ,\theta )=45\alpha^{2}\sin^{2}\varphi +\gamma^{2}\left(3+\sin^{2}\varphi \right),$$

(A5) $$\Phi (S_{∥},I_{∥},\varphi ,\theta )=45\alpha^{2}\cos^{2}\theta \cos^{2}\varphi +\gamma^{2}\left(3+\cos^{2}\theta \cos^{2}\varphi \right),$$

where *α* and *γ* are the mean and the anisotropy of the total polarizability derivative, respectively, with respect to the normal coordinate of the molecule related to the vibration *v_j→k_*. For example, $\Phi (S_{⊥},I_{∥},\varphi ,\theta )$ is the scattering strength function of the perpendicular plane-polarized scattered radiation observed in the direction (*φ*,*θ*), where the Raman scattering is induced by parallel plane-polarized radiation.

The corrected depolarization ratio (*ρ_corr_*) of the band, independent of the collection angle and in the case of perfectly plane-polarized radiation, is defined as the ratio of intensities of the perpendicular and parallel polarized scattered radiation observed from a single point at a zero solid angle in the direction (*φ* = 0, *θ* = π/2). Taking into account the introduced designations, the corrected depolarization ratio is given as:

(A6) $$\rho_{corr}=\frac{\Phi (S_{∥},I_{⊥},\varphi =0,\theta =\pi /2)}{\Phi (S_{⊥},I_{⊥},\varphi =0,\theta =\pi /2)}=\frac{3\gamma^{2}}{45\alpha^{2}+4\gamma^{2}}.$$

According to theoretical calculations reported by Abbate et al. [35], the anisotropy γ of the ν_1_ band of CH_4_ is zero, and hence, *ρ*_corr_(ν_1_) = 0. Besides, α = 0 for the ν_3_ band and *ρ*_corr_(ν_3_) = 0.75, respectively. Let us suppose that the scattered radiation is collected over a finite solid angle Ω and from a region of active molecules characterized by a finite volume *V*. Then, Equation (A1) is given as:

(A7) $$E_{obs}(\Omega ,V)∼E_{in}N_{j}g_{j}\iint_{\Omega ,V}\Phi_{j}(S_{p},I_{p},\varphi ,\theta )d\Omega dV.$$

Let us assume that the exciting radiation in the *V* region is not perfectly plane-polarized due to the introduced polarization distortions and the imperfect polarization of the radiation source. Then, the exciting radiation can be considered as a superposition of two waves with mutually orthogonal polarization:

(A8) $$E_{in}=E_{in}^{⊥}+E_{in}^{∥},$$

where $E_{in}^{⊥}$ and $E_{in}^{∥}$ are the intensities of the perpendicular and parallel plane-polarized exciting radiation, respectively. Therefore, Raman scattering can be described as a superposition of two luminous fluxes, each of which is excited by radiation $E_{in}^{⊥}$ and $E_{in}^{∥}$. In this case, the observed depolarization ratio (*ρ_obs_*) is expressed as follows:

(A9) $$\rho_{obs}=\frac{E_{obs}^{∥}(\Omega ,V)}{E_{obs}^{⊥}(\Omega ,V)}=\frac{\iint_{\Omega ,V}\left[\eta_{1}\Phi (S_{∥},I_{⊥},\varphi ,\theta )+\Phi (S_{∥},I_{∥},\varphi ,\theta )\right]d\Omega dV}{\iint_{\Omega ,V}\left[\eta_{1}\Phi (S_{⊥},I_{⊥},\varphi ,\theta )+\Phi (S_{⊥},I_{∥},\varphi ,\theta )\right]d\Omega dV},$$

where *η*_1_ is the ratio $E_{in}^{⊥}/E_{in}^{∥}$. If the collected radiation from the region *V* undergoes polarization distortions introduced by the collection system, the scattered intensity can also be represented as a superposition on the analogy of Equation (A8). Let us assume that the initially parallel polarized scattered radiation is split into two waves with mutually orthogonal polarization as:

(A10) $$E_{obs}^{∥}\to {\tilde{E}}_{obs∥}^{∥}+{\tilde{E}}_{obs∥}^{⊥},$$

where ${\tilde{E}}_{obs∥}^{∥}$ and ${\tilde{E}}_{obs∥}^{⊥}$ are the intensities of the parallel and the perpendicular plane-polarized observed radiation from the initially parallel polarized scattered radiation, respectively. Then, the separated wave ${\tilde{E}}_{obs∥}^{⊥}$ contributes to the intensity of the perpendicular polarized observed radiation at the end of the collection system. Conversely, the separated wave ${\tilde{E}}_{obs⊥}^{∥}$ from the initially perpendicular polarized scattered radiation contributes to the intensity of the parallel polarized observed radiation. Therefore, Equation (A9) takes the following form:

(A11) $$\rho_{obs}=\frac{{\tilde{E}}_{obs∥}^{∥}(\Omega ,V)+{\tilde{E}}_{obs⊥}^{∥}(\Omega ,V)}{{\tilde{E}}_{obs⊥}^{⊥}(\Omega ,V)+{\tilde{E}}_{obs∥}^{⊥}(\Omega ,V)}.$$

If splitting into two waves occurs in the same ratio for the $E_{obs}^{∥}$ and $E_{obs}^{⊥}$, that is:

(A12) $$\eta_{2}=\frac{{\tilde{E}}_{obs∥}^{∥}}{{\tilde{E}}_{obs∥}^{⊥}}=\frac{{\tilde{E}}_{obs⊥}^{⊥}}{{\tilde{E}}_{obs⊥}^{∥}},$$

then Equation (A11) is expressed as follows:

(A13) $$\rho_{obs}=\frac{\eta_{2}A+B}{\eta_{2}B+A},$$

where:

(A14) $$A=\iint_{\Omega ,V}\left[\eta_{1}\Phi (S_{∥},I_{⊥},\varphi ,\theta )+\Phi (S_{∥},I_{∥},\varphi ,\theta )\right]d\Omega dV,$$

(A15) $$B=\iint_{\Omega ,V}\left[\eta_{1}\Phi (S_{⊥},I_{⊥},\varphi ,\theta )+\Phi (S_{⊥},I_{∥},\varphi ,\theta )\right]d\Omega dV.$$

The Raman spectrometer used in this work detects only those scattered rays that propagate through the collecting lens and are focused into the region of the entrance slit (4 mm × 30 μm) of the spectrometer (see Figure 1 and Figure A1). Lenses used to collect the scattered radiation magnify the image of the object twice. Hence, the effective scattering area is about 2 mm × 15 µm (cross-section of the region *V* in the focal plane of the collecting lens). Therefore, the intensity distribution of the laser beam cross-section and the width (15 µm) of the effective area can be neglected. Hereby, the integration over the solid angle Ω and the region *V* can be replaced by the integration over *φ*, *θ*, and z in Equations (A14) and (A15). Substituting Equation (A6) in Equations (A2)–(A5), the simplified Equation (A13) as a function of the corrected *ρ* can be derived:

(A16) $$A=\eta_{1}\left(CS+\rho_{corr}(U-CS)\right)+\left(CC+\rho_{corr}(U-CC)\right),$$

(A17) $$B=\eta_{1}\left(C+\rho_{corr}(U-C)\right)+\left(S+\rho_{corr}(U-S)\right),$$

where *C*, *S*, *CC*, *CS*, and *U* are triple integrals over the variables *θ*, *φ*, and *z*:

(A18) $$S=4\int_{0}^{\varphi_{\max}}\left(\int_{0}^{z_{\max}}\left(\int_{\theta_{\min}}^{\theta_{\max}}(\sin^{2}\varphi )\sin\theta d\theta \right)dz\right)d\varphi ,$$

(A19) $$C=4\int_{0}^{\varphi_{\max}}\left(\int_{0}^{z_{\max}}\left(\int_{\theta_{\min}}^{\theta_{\max}}(\cos^{2}\varphi )\sin\theta d\theta \right)dz\right)d\varphi ,$$

(A20) $$CC=4\int_{0}^{\varphi_{\max}}\left(\int_{0}^{z_{\max}}\left(\int_{\theta_{\min}}^{\theta_{\max}}(\cos^{2}\theta \cos^{2}\varphi )\sin\theta d\theta \right)dz\right)d\varphi ,$$

(A21) $$CS=4\int_{0}^{\varphi_{\max}}\left(\int_{0}^{z_{\max}}\left(\int_{\theta_{\min}}^{\theta_{\max}}(\cos^{2}\theta \sin^{2}\varphi )\sin\theta d\theta \right)dz\right)d\varphi ,$$

(A22) $$U=4\int_{0}^{\varphi_{\max}}\left(\int_{0}^{z_{\max}}\left(\int_{\theta_{\min}}^{\theta_{\max}}\sin\theta d\theta \right)dz\right)d\varphi ,$$

(A23) $$\theta_{\min}=\arctan\left(\frac{F_{1}}{\cos\varphi \left(\sqrt{0.25D_{1}^{2}-F_{1}^{2}\tan^{2}\varphi }+z\right)}\right),$$

(A24) $$\theta_{\max}=\frac{\pi }{2}+\mathrm{arccot}\left(\frac{F_{1}}{\cos\varphi \left(\sqrt{0.25D_{1}^{2}-F_{1}^{2}\tan^{2}\varphi }-z\right)}\right),$$

(A25) $$z_{\max}=\frac{h_{2}F_{1}}{2F_{2}\cos\varphi },\;\varphi_{\max}=\arctan\left(\frac{D_{1}}{2F_{1}}\right).$$

These expressions are obtained with the proviso that the aperture of the focusing lens does not limit the collection of the scattered radiation from the region of *z* ≠ 0 (*D*_2_ > *D*_1_). Moreover, the aperture and position of the exit window of the gas cell are neglected for the same reason.

As discussed in Section 3.3., the polarization scrambling effects of scattered radiation propagating through the path of W_1_ → W_3_ → Polarizer or W_1_ → W_2_ → Polarizer are similar (see Figure 6). Thus, the approximate equality *η*_1_ ≈ *η*_2_ ≥ 1000 holds in Equations (A13), (A16) and (A17). In other words, the perpendicular plane-polarized laser radiation will contain 1 part of the parallel polarized light to more than 1000 perpendicular polarized parts, at the end of the collection system. Figure A2 shows the deviation of the *ρ_obs_* from the *ρ_corr_* as a function of *ρ_corr_*, parameter *η*, and the full collection angle (2*φ*_max_). These values are calculated using Equations (A13) and (A16)–(A25), and the parameters from Table 1. As expected, the collection of radiation using the lens with a larger f-number leads to a greater deviation of the *ρ*. However, the deviation of the *ρ* tends to a value of 1/*η* in the vicinity of the zero-collection angle. This deviation is caused by the splitting of the plane-polarized scattered radiation as 1 to *η* due to the polarization scrambling effect, even in the case of collecting from a single point at a zero solid angle. In turn, the attenuation of the polarization scrambling effect leads to less deviation of the *ρ* at a fixed collection angle. The deviation of the *ρ* is approximately 0.001 at the *η* = 1000 and 2*φ*_max_ = 14.25°.

## References

[1] Petrov D.V. (2016). Multipass optical system for a Raman gas spectrometer. Appl. Opt..

[2] Yang D., Guo J., Liu Q., Luo Z., Yan J., Zheng R. (2016). Highly sensitive Raman system for dissolved gas analysis in water. Appl. Opt..

[3] Wen C., Huang X., Shen C. (2020). Multiple-pass enhanced Raman spectroscopy for fast industrial trace gas detection and process control. J. Raman Spectrosc..

[4] Guo J., Luo Z., Liu Q., Yang D., Dong H., Huang S., Kong A., Wu L. (2021). High-Sensitivity Raman Gas Probe for In Situ Multi-component Gas Detection. Sensors.

[5] Hanf S., Keiner R., Yan D., Popp J., Frosch T. (2014). Fiber-Enhanced Raman Multigas Spectroscopy: A Versatile Tool for Environmental Gas Sensing and Breath Analysis. Anal. Chem..

[6] Knebl A., Yan D., Popp J., Frosch T. (2018). Fiber enhanced Raman gas spectroscopy. Trends Anal. Chem..

[7] Sieburg A., Knebl A., Jacob J.M., Frosch T. (2019). Characterization of fuel gases with fiber-enhanced Raman spectroscopy. Anal. Bioanal. Chem..

[8] Knebl A., Domes R., Yan D., Popp J., Trumbore S., Frosch T. (2019). Fiber-Enhanced Raman Gas Spectroscopy for ^18^O–^13^C-Labeling Experiments. Anal. Chem..

[9] Buldakov M.A., Matrosov I.I., Petrov D.V., Tikhomirov A.A. (2012). Raman gas-analyzer for analyzing environmental and technogenic gas media. Atmos. Ocean. Opt..

[10] Petrov D.V., Matrosov I.I., Zaripov A.R. (2018). Determination of atmospheric carbon dioxide concentration using Raman spectroscopy. J. Mol. Spectrosc..

[11] Petrov D.V., Matrosov I.I., Tikhomirov A.A. (2015). High-Sensitivity Spontaneous Raman Spectrometer for Gaseous Media. J. Appl. Spectrosc..

[12] Petrov D.V., Matrosov I.I., Kostenko M.A. (2021). Possibilities of measuring the exhaled air composition using Raman spectroscopy. Quantum Electron..

[13] Chow K.K., Short M., Lam S., McWilliams A., Zeng H. (2014). A Raman cell based on hollow core photonic crystal fiber for human breath analysis. Med. Phys..

[14] Keiner R., Frosch T., Massad T., Trumbore S., Popp J. (2014). Enhanced Raman multigas sensing—A novel tool for control and analysis of ^13^CO_2_ labeling experiments in environmental research. Analyst.

[15] Velez J.G., Muller A. (2020). Trace gas sensing using diode-pumped collinearly detected spontaneous Raman scattering enhanced by a multipass cell. Opt. Lett..

[16] Velez J.S.G., Muller A. (2021). Spontaneous Raman scattering at trace gas concentrations with a pressurized external multipass cavity. Meas. Sci. Technol..

[17] Yakovlev S., Sadovnikov S., Kharchenko O., Kravtsova N. (2020). Remote Sensing of Atmospheric Methane with IR OPO Lidar System. Atmosphere.

[18] Buszewski B., Kęsy M., Ligor T., Amann A. (2007). Human exhaled air analytics: Biomarkers of diseases. Biomed. Chromatogr..

[19] Mazzatenta A., Di Giulio C., Pokorski M. (2013). Pathologies currently identified by exhaled biomarkers. Respir. Physiol. Neurobiol..

[20] Schrötter H.W., Klöckner H.W., Weber A. (1979). Raman Scattering Cross Sections in Gases and Liquids. Raman Spectroscopy of Gases and Liquids.

[21] Stephenson D.A. (1974). Raman cross sections of selected hydrocarbons and freons. J. Quant. Spectrosc. Radiat. Transf..

[22] Le V.H., Tarantola A., Caumon M.C. (2021). Interpretation of the pressure-induced Raman frequency shift of the ν_1_ stretching bands of CH_4_ and N_2_ within CH_4_-CO_2_, N_2_-CO_2_ and CH_4_-N_2_ binary mixtures. Phys. Chem. Chem. Phys..

[23] Ridder M., Suvernev A.A., Dreier T. (1996). Collision effects in nitrogen and methane coherent anti-Stokes Raman isotropic Q-branch spectra at high densities. J. Chem. Phys..

[24] Seitz J.C., Pasteris J.D., Chou I.-M. (1993). Raman spectroscopic characterization of gas mixtures. I. Quantitative composition and pressure determination of CH_4_, N_2_ and their mixtures. Am. J. Sci..

[25] Seitz J.C., Pasteris J.D., Chou I.-M. (1996). Raman spectroscopic characterization of gas mixtures. II. Quantitative composition and pressure determination of the CO_2_-CH_4_ system. Am. J. Sci..

[26] Pieroni D., Hartmann J.-M., Chaussard F., Michaut X., Gabard T., Saint-Loup R., Berger H., Champion J.-P. (2000). Experimental and theoretical study of line mixing in methane spectra. III. The Q branch of the Raman ν_1_ band. J. Chem. Phys..

[27] Sublett D.M., Sendula E., Lamadrid H.M., Steele-MacInnis M., Spiekermann G., Bodnar R.J. (2021). Raman spectral behavior of N_2_, CO_2_, and CH_4_ in N_2_–CO_2_–CH_4_ gas mixtures from 22 °C to 200 °C and 10 to 500 bars, with application to other gas mixtures. J. Raman Spectrosc..

[28] Sublett D.M., Sendula E., Lamadrid H., Steele-MacInnis M., Spiekermann G., Burruss R.C., Bodnar R.J. (2020). Shift in the Raman symmetric stretching band of N_2_, CO_2_, and CH_4_ as a function of temperature, pressure, and density. J. Raman Spectrosc..

[29] Petrov D.V. (2017). Pressure dependence of peak positions, half widths, and peak intensities of methane Raman bands (ν_2_, 2ν_4_, ν_1_, ν_3_, and 2ν_2_). J. Raman Spectrosc..

[30] Petrov D.V. (2018). Raman spectrum of methane in nitrogen, carbon dioxide, hydrogen, ethane, and propane environments. Spectrochim. Acta—Part A Mol. Biomol. Spectrosc..

[31] Jourdanneau E., Chaussard F., Saint-Loup R., Gabard T., Berger H. (2005). The methane Raman spectrum from 1200 to 5500 cm^−1^: A first step toward temperature diagnostic using methane as a probe molecule in combustion systems. J. Mol. Spectrosc..

[32] Jourdanneau E., Gabard T., Chaussard F., Saint-Loup R., Berger H., Bertseva E., Grisch F. (2007). CARS methane spectra: Experiments and simulations for temperature diagnostic purposes. J. Mol. Spectrosc..

[33] Tanichev A.S., Petrov D.V. (2022). Simulation of ν_2_ Raman band of methane as a function of pressure. J. Raman Spectrosc..

[34] Boudon V., Champion J.-P., Gabard T., Loëte M., Rotger M., Wenger C., Quack M., Merkt F. (2011). Spherical Top Theory and Molecular Spectra. Handbook of High-Resolution Spectroscopy.

[35] Abbate S., Gussoni M., Zerbi G. (1978). Raman intensities of methanes from electrooptical parameters. J. Mol. Spectrosc..

[36] Hyodo S. (1991). Fluctuation of Local Field and Depolarization Ratio of the ν_1_ Raman Line of Carbon Tetrachloride in Carbon Disulfide Solution. Bull. Chem. Soc. Jpn..

[37] Buldakov M.A., Cherepanov V.N., Korolev B.V., Matrosov I.I. (2003). Role of intramolecular interactions in Raman spectra of N_2_ and O_2_ molecules. J. Mol. Spectrosc..

[38] Wang P.G., Ziegler L.D. (1993). Polarization analysis of the 266-nm excited resonance Raman spectrum of methyl iodide. J. Phys. Chem..

[39] Yu Y., Lin K., Zhou X., Wang H., Liu S., Ma X. (2007). Precise measurement of the depolarization ratio from photoacoustic Raman spectroscopy. J. Raman Spectrosc..

[40] Rose E.J., Whitewolf E., Baglin F.G. (1992). Isothermal density tuning of the depolarization ratios from the ν_1_ mode of methane. J. Chem. Phys..

[41] Wright M., Murphy T., Baglin F.G. (1994). Isosteric and isothermal studies of the Raman depolarization ratios in an argon-methane mixture at 298 K and 323 K. Mol. Phys..

[42] Petrov D. (2020). Depolarization Ratios of Methane Raman Bands as a Function of Pressure. Molecules.

[43] Rose E.J., Baglin F.G. (1994). Depolarization-density tuning in supracritical solutions of methane-carbon dioxide. Mol. Phys..

[44] Rose E.J., Baglin F.G. (1994). Isothermal Raman depolarization ratios of supracritical carbon monoxide at pressures between 20 and 2400 bar. J. Raman Spectrosc..

[45] Baglin F.G., Sweitzer S., Stanbery W. (1996). Raman light scattering from supracritical binary fluid mixtures: CH_4_/CF_4_. J. Chem. Phys..

[46] Lemmon E.W., McLinden M.O., Friend D.G., Linstrom P.J., Mallard W.G. (2021). Thermophysical Properties of Fluid Systems. NIST Chemistry WebBook, NIST Standard Reference Database Number 69.

[47] Brunsgaard Hansen S., Berg R.W., Stenby E.H. (2004). Upgrade of a Raman spectrometer. Appl. Spectrosc. Rev..

[48] Berger H. (1977). Raman spectrum of ^12^CH_4_ between 2850 and 3100 cm^−1^. J. Mol. Spectrosc..

[49] Qiu Y., Wang X.-L., Liu X., Cao J., Liu Y.-F., Xi B.-B., Gao W.-L. (2020). In situ Raman spectroscopic quantification of CH_4_–CO_2_ mixture: Application to fluid inclusions hosted in quartz veins from the Longmaxi Formation shales in Sichuan Basin, southwestern China. Pet. Sci..

[50] Fang J., Chou I.-M., Chen Y. (2018). Quantitative Raman spectroscopic study of the H_2_–CH_4_ gaseous system. J. Raman Spectrosc..

[51] Lamadrid H.M., Steele-MacInnis M., Bodnar R.J. (2018). Relationship between Raman spectral features and fugacity in mixtures of gases. J. Raman Spectrosc..

[52] Ba Y.A., Wenger C., Surleau R., Boudon V., Rotger M., Daumont L., Bonhommeau D.A., Tyuterev V.G., Dubernet M.-L. (2013). MeCaSDa and ECaSDa: Methane and ethene calculated spectroscopic databases for the virtual atomic and molecular data centre. J. Quant. Spectrosc. Radiat. Transf..

[53] Montero S., Bermejo D. (1976). Electro-optical parameters and Raman intensities of CH_4_, CH_3_D, CH_2_D_2_, CHD_3_ and CD_4_. Mol. Phys..

[54] Applequist J., Quicksall C.O. (1977). Calculation of Raman scattering parameters for methane and halomethanes from an atom dipole interaction model. J. Chem. Phys..

[55] Cantor D.M., Schroeder J., Jonas J. (1975). Polarization Scrambling by Optical Windows Used for Light Scattering Experiments at High Pressures. Appl. Spectrosc..

[56] Perry S., Sharko P.T., Jonas J. (1983). Technique for Measuring the Amount of Pressure-Induced Polarization Scrambling by Optical Windows in High Pressure Light Scattering Cells. Appl. Spectrosc..

[57] Schlösser M., James T.M., Fischer S., Lewis R.J., Bornschein B., Telle H.H. (2013). Evaluation method for Raman depolarization measurements including geometrical effects and polarization aberrations. J. Raman Spectrosc..

[58] James T.M., Schlösser M., Fischer S., Sturm M., Bornschein B., Lewis R.J., Telle H.H. (2013). Accurate depolarization ratio measurements for all diatomic hydrogen isotopologues. J. Raman Spectrosc..

[59] Teboul V., Godet J.L., Le Duff Y. (1992). Collection Angle Dependence of the Depolarization Ratio in Light-Scattering Experiments. Appl. Spectrosc..

[60] Long D.A. (2002). The Raman Effect: A Unified Treatment of the Theory of Raman Scattering by Molecules.

